# Triaqua­(2,2′-bipyridine-κ^2^ *N*,*N*′)(5-nitro­isophthalato-κ*O* ^1^)zinc(II) monohydrate

**DOI:** 10.1107/S1600536808035174

**Published:** 2008-11-08

**Authors:** Lujiang Hao, Xia Liu

**Affiliations:** a College of Food and Biological Engineering, Shandong Institute of Light Industry, Jinan 250353, People’s Republic of China; b Maize Research Institute, Shandong Academy of Agricultural Science, Jinan 250100, People’s Republic of China

## Abstract

In the title compound, [Zn(C_8_H_3_NO_6_)(C_10_H_8_N_2_)(H_2_O)_3_]·H_2_O, the Zn^II^ cation is hexa­coordinated by a chelating 2,2′-bipyridine ligand, one carboxyl­ate O atom from a 5-nitro­isophthalate dianion and three water mol­ecules in a slightly distorted octa­hedral geometry. The structure contains isolated neutral complexes, in contrast to coordination polymers formed by Mn^II^, Co^II^ and Cu^II^ with the same ligand set. An extensive network of hydrogen bonds is formed between the water mol­ecules and the carboxyl­ate groups.

## Related literature

For related coordination polymers formed with the same ligand set and Mn^II^, Co^II^ or Cu^II^, see: Xiao *et al.* (2005 ▶); Xie *et al.* (2005 ▶, 2006 ▶). For other examples of transition-metal complexes containing benzene carboxyl­ates and pyridine-based ligands, see: Kim *et al.* (2001 ▶).
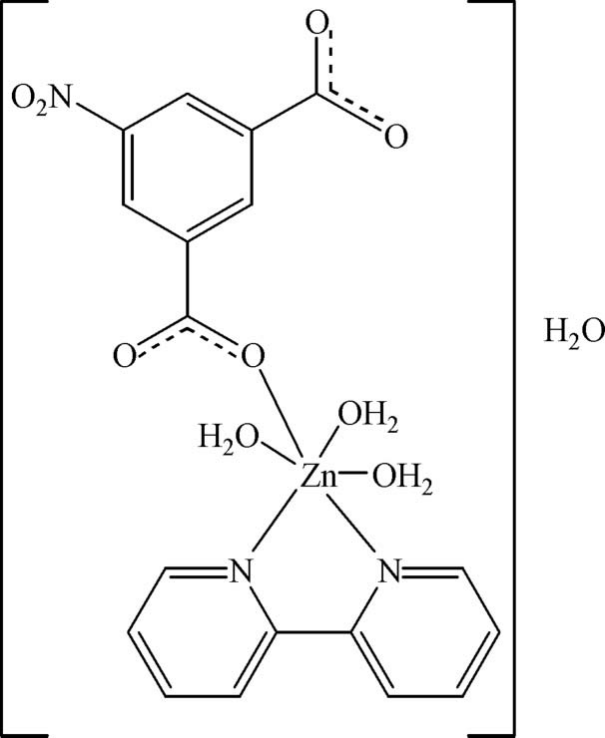


## Experimental

### 

#### Crystal data

[Zn(C_8_H_3_NO_6_)(C_10_H_8_N_2_)(H_2_O)_3_]·H_2_O
*M* *_r_* = 502.73
Triclinic, 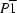

*a* = 7.5200 (10) Å
*b* = 10.6700 (15) Å
*c* = 12.8300 (15) Å
α = 90.024 (10)°
β = 87.670 (10)°
γ = 74.720 (10)°
*V* = 992.2 (2) Å^3^
*Z* = 2
Mo *K*α radiation
μ = 1.30 mm^−1^
*T* = 293 (2) K
0.32 × 0.28 × 0.22 mm

#### Data collection

Bruker APEXII CCD diffractometer
Absorption correction: multi-scan (*SADABS*; Bruker, 2001 ▶) *T*_min_ = 0.592, *T*_max_ = 0.747
5594 measured reflections
3801 independent reflections
3240 reflections with *I* > 2σ(*I*)
*R*_int_ = 0.016

#### Refinement

*R*[*F* ^2^ > 2σ(*F* ^2^)] = 0.051
*wR*(*F* ^2^) = 0.147
*S* = 1.06
3801 reflections
289 parameters
H-atom parameters constrained
Δρ_max_ = 1.13 e Å^−3^
Δρ_min_ = −0.72 e Å^−3^

### 

Data collection: *APEX2* (Bruker, 2004 ▶); cell refinement: *SAINT-Plus* (Bruker, 2001 ▶); data reduction: *SAINT-Plus*; program(s) used to solve structure: *SHELXS97* (Sheldrick, 2008 ▶); program(s) used to refine structure: *SHELXL97* (Sheldrick, 2008 ▶); molecular graphics: *SHELXTL* (Sheldrick, 2008 ▶); software used to prepare material for publication: *SHELXTL*.

## Supplementary Material

Crystal structure: contains datablocks global, I. DOI: 10.1107/S1600536808035174/bi2306sup1.cif

Structure factors: contains datablocks I. DOI: 10.1107/S1600536808035174/bi2306Isup2.hkl

Additional supplementary materials:  crystallographic information; 3D view; checkCIF report

## Figures and Tables

**Table 1:** Hydrogen-bond geometry (Å, °)

*D*—H⋯*A*	*D*—H	H⋯*A*	*D*⋯*A*	*D*—H⋯*A*
O7—H2*W*⋯O3^i^	0.84	1.96	2.776 (4)	165
O7—H1*W*⋯O10^ii^	0.84	1.78	2.607 (4)	168
O8—H3*W*⋯O5	0.84	1.94	2.715 (4)	153
O8—H4*W*⋯O3^iii^	0.84	1.89	2.721 (4)	172
O9—H5*W*⋯O3^iv^	0.84	1.94	2.727 (4)	156
O10—H8*W*⋯O4^vi^	0.84	1.79	2.631 (4)	180
O10—H7*W*⋯O5	0.84	1.87	2.713 (5)	180

Symmetry codes: (i) 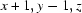; (ii) 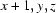; (iii) 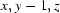; (iv) 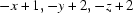; (v) 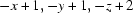; (vi) 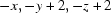.

**Fig. 1.**
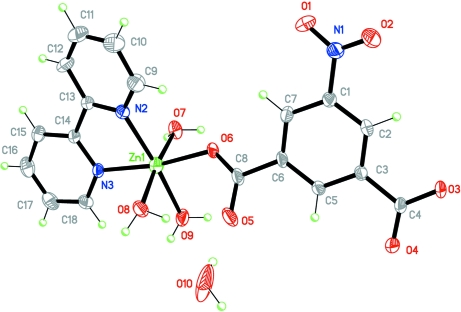
Molecular structure of the title compound showing displacement ellipsoids at 50% probability for non-H atoms.

## supplementary crystallographic information

### Comment

In recent years, carboxylic acids have been widely used in materials science as polydentate ligands which can coordinate to transition-metal or rare-earth cations to yield complexes with interesting or useful properties. For example, Kim *et al.* (2001) have focused on the syntheses of transition-metal complexes containing benzene carboxylate and rigid aromatic pyridine ligands in order to study their electronic conductivity and magnetic properties. The importance of transition-metal dicarboxylate complexes motivated us to pursue synthetic strategies for these compounds, using 5-nitroisophthalic acid as a polydentate ligand.

### Experimental

A mixture of zinc dichloride (0.5 mmol), 2,2'-bipyridine (0.5 mmol), and 5-nitroisophthalic acid (0.5 mmol) in H_2_O (8 ml) and ethanol (8 ml) was sealed in a 25 ml Teflon-lined stainless steel autoclave and kept at 413 K for three days. Colourless crystals were obtained after cooling to room temperature with a yield of 27%. Elemental analysis calculated: C 42.97, H 3.78, N 9.55%; found: C 42.86, H 3.76, N 9.51%.

### Refinement

The H atoms of the water molecule were located from difference density maps. The O—H bonds were normalised to 0.84 Å, and the H atoms were then allowed to ride on the parent O atom with *U*_iso_(H) = 1.5*U*_eq_(O). All other H atoms were placed in calculated positions with a C—H bond distance of 0.93 Å and refined as riding with *U*_iso_(H) = 1.2*U*_eq_(C).

### Crystal data

**Table:**

[Zn(C_8_H_3_NO_6_)(C_10_H_8_N_2_)(H_2_O)_3_]·H_2_O	*Z* = 2
*M**_r_* = 502.73	*F*_000_ = 516
Triclinic, *P*1	*D*_x_ = 1.683 Mg m^−^^3^
Hall symbol: -P 1	Mo *K*α radiation λ = 0.71073 Å
*a* = 7.5200 (10) Å	Cell parameters from 3801 reflections
*b* = 10.6700 (15) Å	θ = 1.6–26.0º
*c* = 12.8300 (15) Å	µ = 1.30 mm^−^^1^
α = 90.024 (10)º	*T* = 293 (2) K
β = 87.670 (10)º	Block, colorless
γ = 74.720 (10)º	0.32 × 0.28 × 0.22 mm
*V* = 992.2 (2) Å^3^	

### Data collection

**Table:**

Bruker APEXII CCD diffractometer	3801 independent reflections
Radiation source: fine-focus sealed tube	3240 reflections with *I* > 2σ(*I*)
Monochromator: graphite	*R*_int_ = 0.016
*T* = 293(2) K	θ_max_ = 26.0º
φ and ω scans	θ_min_ = 1.6º
Absorption correction: multi-scan(SADABS; Bruker, 2001)	*h* = −9→9
*T*_min_ = 0.592, *T*_max_ = 0.747	*k* = −13→13
5594 measured reflections	*l* = 0→15

### Refinement

**Table:**

Refinement on *F*^2^	Secondary atom site location: difference Fourier map
Least-squares matrix: full	Hydrogen site location: inferred from neighbouring sites
*R*[*F*^2^ > 2σ(*F*^2^)] = 0.051	H-atom parameters constrained
*wR*(*F*^2^) = 0.147	*w* = 1/[σ^2^(*F*_o_^2^) + (0.0817*P*)^2^ + 1.7563*P*] where *P* = (*F*_o_^2^ + 2*F*_c_^2^)/3
*S* = 1.06	(Δ/σ)_max_ < 0.001
3801 reflections	Δρ_max_ = 1.13 e Å^−^^3^
289 parameters	Δρ_min_ = −0.72 e Å^−^^3^
Primary atom site location: structure-invariant direct methods	Extinction correction: none

### Special details

**Table:**

Geometry. All esds (except the esd in the dihedral angle between two l.s. planes) are estimated using the full covariance matrix. The cell esds are taken into account individually in the estimation of esds in distances, angles and torsion angles; correlations between esds in cell parameters are only used when they are defined by crystal symmetry. An approximate (isotropic) treatment of cell esds is used for estimating esds involving l.s. planes.
Refinement. Refinement of F^2^ against ALL reflections. The weighted R-factor wR and goodness of fit S are based on F^2^, conventional R-factors R are based on F, with F set to zero for negative F^2^. The threshold expression of F^2^ > 2sigma(F^2^) is used only for calculating R-factors(gt) etc. and is not relevant to the choice of reflections for refinement. R-factors based on F^2^ are statistically about twice as large as those based on F, and R- factors based on ALL data will be even larger.

### Fractional atomic coordinates and isotropic or equivalent isotropic displacement parameters (Å^2^)

**Table:**

	*x*	*y*	*z*	*U*_iso_*/*U*_eq_	
Zn1	0.72904 (6)	0.47118 (4)	0.78573 (3)	0.02670 (18)	
C1	0.5256 (5)	1.0550 (3)	0.6738 (3)	0.0200 (7)	
C2	0.4383 (5)	1.1472 (3)	0.7462 (3)	0.0204 (7)	
H2A	0.4166	1.2351	0.7314	0.024*	
C3	0.3825 (5)	1.1075 (3)	0.8421 (3)	0.0185 (7)	
C4	0.2892 (5)	1.2039 (3)	0.9251 (3)	0.0192 (7)	
C5	0.4090 (5)	0.9779 (3)	0.8610 (3)	0.0206 (7)	
H5A	0.3675	0.9513	0.9243	0.025*	
C6	0.4968 (5)	0.8863 (3)	0.7869 (3)	0.0212 (7)	
C7	0.5604 (5)	0.9247 (3)	0.6925 (3)	0.0230 (7)	
H7A	0.6248	0.8637	0.6433	0.028*	
C8	0.5179 (5)	0.7461 (3)	0.8070 (3)	0.0258 (8)	
C9	0.7336 (6)	0.5092 (4)	0.5475 (3)	0.0323 (9)	
H9A	0.6630	0.5932	0.5630	0.039*	
C10	0.7887 (7)	0.4771 (5)	0.4446 (3)	0.0434 (11)	
H10A	0.7530	0.5368	0.3917	0.052*	
C11	0.8961 (8)	0.3562 (5)	0.4235 (3)	0.0491 (13)	
H11A	0.9383	0.3321	0.3554	0.059*	
C12	0.9428 (7)	0.2692 (4)	0.5027 (3)	0.0396 (11)	
H12A	1.0183	0.1861	0.4890	0.048*	
C13	0.8759 (5)	0.3064 (3)	0.6038 (3)	0.0216 (7)	
C14	0.9011 (5)	0.2164 (3)	0.6926 (3)	0.0190 (7)	
C15	0.9850 (5)	0.0861 (4)	0.6817 (3)	0.0279 (8)	
H15A	1.0429	0.0516	0.6187	0.033*	
C16	0.9811 (6)	0.0076 (4)	0.7667 (4)	0.0355 (10)	
H16A	1.0358	−0.0812	0.7611	0.043*	
C17	0.8971 (6)	0.0598 (4)	0.8594 (3)	0.0326 (9)	
H17A	0.8908	0.0071	0.9165	0.039*	
C18	0.8232 (5)	0.1903 (4)	0.8660 (3)	0.0267 (8)	
H18A	0.7700	0.2267	0.9295	0.032*	
N1	0.5836 (5)	1.0974 (3)	0.5725 (2)	0.0275 (7)	
N2	0.7767 (4)	0.4257 (3)	0.6256 (2)	0.0215 (6)	
N3	0.8239 (4)	0.2684 (3)	0.7848 (2)	0.0192 (6)	
O1	0.6969 (4)	1.0197 (3)	0.5172 (2)	0.0373 (7)	
O2	0.5184 (5)	1.2084 (3)	0.5476 (2)	0.0445 (8)	
O3	0.2776 (4)	1.3214 (2)	0.9070 (2)	0.0255 (6)	
O4	0.2275 (4)	1.1635 (3)	1.0056 (2)	0.0337 (7)	
O5	0.3987 (5)	0.7173 (3)	0.8669 (3)	0.0524 (10)	
O6	0.6527 (4)	0.6677 (2)	0.7630 (2)	0.0254 (6)	
O7	1.0013 (4)	0.4817 (3)	0.7943 (2)	0.0272 (6)	
H1W	1.0042	0.5549	0.8172	0.041*	
H2W	1.0698	0.4272	0.8325	0.041*	
O8	0.4497 (4)	0.4721 (2)	0.7915 (2)	0.0273 (6)	
H3W	0.3989	0.5451	0.8186	0.041*	
H4W	0.4061	0.4197	0.8264	0.041*	
O9	0.7038 (4)	0.4909 (3)	0.9526 (2)	0.0347 (7)	
H5W	0.7188	0.5586	0.9802	0.052*	
H6W	0.6784	0.4397	0.9970	0.052*	
O10	0.0498 (5)	0.6904 (4)	0.8813 (5)	0.113 (3)	
H7W	0.1579	0.6986	0.8768	0.169*	
H8W	−0.0387	0.7370	0.9175	0.169*	

### Atomic displacement parameters (Å^2^)

**Table:**

	*U*^11^	*U*^22^	*U*^33^	*U*^12^	*U*^13^	*U*^23^
Zn1	0.0308 (3)	0.0205 (3)	0.0280 (3)	−0.00584 (19)	0.00216 (18)	−0.00145 (17)
C1	0.0244 (18)	0.0183 (17)	0.0177 (16)	−0.0059 (14)	−0.0015 (14)	−0.0002 (13)
C2	0.0244 (18)	0.0132 (16)	0.0245 (18)	−0.0060 (13)	−0.0034 (14)	0.0019 (13)
C3	0.0185 (17)	0.0152 (16)	0.0220 (17)	−0.0050 (13)	0.0002 (13)	−0.0002 (13)
C4	0.0197 (17)	0.0125 (16)	0.0241 (17)	−0.0019 (13)	−0.0012 (14)	−0.0004 (13)
C5	0.0203 (17)	0.0149 (16)	0.0257 (18)	−0.0037 (13)	0.0036 (14)	0.0015 (13)
C6	0.0199 (17)	0.0101 (16)	0.0322 (19)	−0.0021 (13)	0.0017 (14)	0.0000 (14)
C7	0.0252 (18)	0.0167 (17)	0.0260 (18)	−0.0039 (14)	0.0017 (15)	−0.0051 (14)
C8	0.0234 (19)	0.0114 (16)	0.042 (2)	−0.0038 (14)	0.0057 (16)	−0.0009 (15)
C9	0.042 (2)	0.025 (2)	0.027 (2)	−0.0042 (17)	−0.0037 (17)	0.0066 (16)
C10	0.068 (3)	0.041 (3)	0.023 (2)	−0.016 (2)	−0.006 (2)	0.0102 (18)
C11	0.085 (4)	0.046 (3)	0.019 (2)	−0.023 (3)	0.010 (2)	−0.0022 (19)
C12	0.060 (3)	0.032 (2)	0.024 (2)	−0.010 (2)	0.016 (2)	−0.0078 (17)
C13	0.0265 (19)	0.0175 (17)	0.0211 (17)	−0.0069 (14)	0.0033 (14)	−0.0011 (13)
C14	0.0215 (17)	0.0160 (16)	0.0197 (17)	−0.0053 (13)	−0.0003 (13)	−0.0003 (13)
C15	0.031 (2)	0.0176 (18)	0.032 (2)	−0.0019 (15)	0.0039 (16)	−0.0042 (15)
C16	0.040 (2)	0.0165 (19)	0.048 (3)	−0.0037 (17)	−0.006 (2)	0.0033 (17)
C17	0.038 (2)	0.027 (2)	0.036 (2)	−0.0121 (18)	−0.0078 (18)	0.0127 (17)
C18	0.035 (2)	0.0255 (19)	0.0212 (18)	−0.0117 (16)	0.0004 (15)	0.0040 (15)
N1	0.0378 (19)	0.0274 (17)	0.0202 (15)	−0.0138 (15)	−0.0002 (14)	−0.0003 (13)
N2	0.0273 (16)	0.0189 (15)	0.0186 (14)	−0.0070 (12)	−0.0002 (12)	0.0019 (11)
N3	0.0244 (15)	0.0128 (13)	0.0200 (14)	−0.0045 (11)	−0.0006 (12)	−0.0011 (11)
O1	0.0459 (18)	0.0380 (17)	0.0252 (14)	−0.0082 (14)	0.0138 (13)	−0.0063 (12)
O2	0.075 (2)	0.0262 (16)	0.0294 (16)	−0.0094 (15)	0.0061 (15)	0.0088 (12)
O3	0.0364 (15)	0.0108 (12)	0.0276 (13)	−0.0042 (10)	0.0049 (11)	−0.0013 (10)
O4	0.0486 (18)	0.0168 (13)	0.0314 (15)	−0.0041 (12)	0.0177 (13)	0.0001 (11)
O5	0.0441 (19)	0.0160 (14)	0.095 (3)	−0.0102 (13)	0.0398 (19)	−0.0053 (15)
O6	0.0282 (14)	0.0084 (11)	0.0365 (15)	−0.0011 (10)	0.0110 (11)	−0.0008 (10)
O7	0.0257 (14)	0.0190 (13)	0.0376 (15)	−0.0061 (10)	−0.0077 (11)	0.0022 (11)
O8	0.0232 (13)	0.0186 (13)	0.0402 (15)	−0.0065 (10)	0.0059 (11)	0.0013 (11)
O9	0.065 (2)	0.0230 (14)	0.0190 (13)	−0.0171 (14)	0.0029 (13)	−0.0040 (10)
O10	0.036 (2)	0.074 (3)	0.229 (7)	−0.025 (2)	0.051 (3)	−0.107 (4)

### Geometric parameters (Å, °)

**Table:**

Zn1—O6	2.047 (2)	C11—C12	1.370 (7)
Zn1—O7	2.087 (3)	C11—H11A	0.930
Zn1—N3	2.092 (3)	C12—C13	1.391 (5)
Zn1—O8	2.096 (3)	C12—H12A	0.930
Zn1—N2	2.105 (3)	C13—N2	1.318 (5)
Zn1—O9	2.148 (3)	C13—C14	1.475 (5)
C1—C2	1.368 (5)	C14—N3	1.349 (4)
C1—C7	1.369 (5)	C14—C15	1.371 (5)
C1—N1	1.463 (5)	C15—C16	1.380 (6)
C2—C3	1.386 (5)	C15—H15A	0.930
C2—H2A	0.930	C16—C17	1.370 (6)
C3—C5	1.367 (5)	C16—H16A	0.930
C3—C4	1.497 (5)	C17—C18	1.357 (6)
C4—O4	1.240 (4)	C17—H17A	0.930
C4—O3	1.255 (4)	C18—N3	1.335 (5)
C5—C6	1.380 (5)	C18—H18A	0.930
C5—H5A	0.930	N1—O2	1.204 (5)
C6—C7	1.385 (5)	N1—O1	1.222 (4)
C6—C8	1.486 (5)	O7—H1W	0.840
C7—H7A	0.930	O7—H2W	0.840
C8—O6	1.245 (4)	O8—H3W	0.840
C8—O5	1.256 (5)	O8—H4W	0.840
C9—N2	1.334 (5)	O9—H5W	0.840
C9—C10	1.382 (6)	O9—H6W	0.840
C9—H9A	0.930	O10—H7W	0.840
C10—C11	1.350 (7)	O10—H8W	0.840
C10—H10A	0.930		
			
O6—Zn1—O7	88.46 (11)	C10—C11—C12	119.8 (4)
O6—Zn1—N3	170.99 (11)	C10—C11—H11A	120.1
O7—Zn1—N3	89.04 (11)	C12—C11—H11A	120.1
O6—Zn1—O8	89.13 (10)	C11—C12—C13	119.3 (4)
O7—Zn1—O8	174.03 (10)	C11—C12—H12A	120.4
N3—Zn1—O8	94.16 (11)	C13—C12—H12A	120.4
O6—Zn1—N2	94.11 (11)	N2—C13—C12	121.2 (4)
O7—Zn1—N2	89.58 (11)	N2—C13—C14	115.1 (3)
N3—Zn1—N2	77.22 (11)	C12—C13—C14	123.6 (3)
O8—Zn1—N2	96.04 (11)	N3—C14—C15	121.4 (3)
O6—Zn1—O9	93.40 (11)	N3—C14—C13	115.7 (3)
O7—Zn1—O9	87.99 (12)	C15—C14—C13	122.8 (3)
N3—Zn1—O9	95.16 (11)	C14—C15—C16	118.2 (4)
O8—Zn1—O9	86.70 (12)	C14—C15—H15A	120.9
N2—Zn1—O9	172.05 (11)	C16—C15—H15A	120.9
C2—C1—C7	122.4 (3)	C17—C16—C15	120.4 (4)
C2—C1—N1	118.8 (3)	C17—C16—H16A	119.8
C7—C1—N1	118.8 (3)	C15—C16—H16A	119.8
C1—C2—C3	119.0 (3)	C18—C17—C16	118.4 (4)
C1—C2—H2A	120.5	C18—C17—H17A	120.8
C3—C2—H2A	120.5	C16—C17—H17A	120.8
C5—C3—C2	119.6 (3)	N3—C18—C17	122.5 (4)
C5—C3—C4	119.0 (3)	N3—C18—H18A	118.8
C2—C3—C4	121.3 (3)	C17—C18—H18A	118.8
O4—C4—O3	124.6 (3)	O2—N1—O1	122.8 (3)
O4—C4—C3	118.5 (3)	O2—N1—C1	118.3 (3)
O3—C4—C3	117.0 (3)	O1—N1—C1	118.9 (3)
C3—C5—C6	120.6 (3)	C13—N2—C9	118.5 (3)
C3—C5—H5A	119.7	C13—N2—Zn1	115.1 (2)
C6—C5—H5A	119.7	C9—N2—Zn1	126.0 (3)
C5—C6—C7	120.3 (3)	C18—N3—C14	119.1 (3)
C5—C6—C8	119.9 (3)	C18—N3—Zn1	126.6 (2)
C7—C6—C8	119.8 (3)	C14—N3—Zn1	114.2 (2)
C1—C7—C6	118.0 (3)	C8—O6—Zn1	125.6 (2)
C1—C7—H7A	121.0	Zn1—O7—H1W	110.4
C6—C7—H7A	121.0	Zn1—O7—H2W	116.7
O6—C8—O5	125.9 (3)	H1W—O7—H2W	105.6
O6—C8—C6	117.0 (3)	Zn1—O8—H3W	102.2
O5—C8—C6	117.1 (3)	Zn1—O8—H4W	124.1
N2—C9—C10	123.2 (4)	H3W—O8—H4W	104.6
N2—C9—H9A	118.4	Zn1—O9—H5W	118.6
C10—C9—H9A	118.4	Zn1—O9—H6W	129.1
C11—C10—C9	117.9 (4)	H5W—O9—H6W	112.3
C11—C10—H10A	121.1	H7W—O10—H8W	126.1
C9—C10—H10A	121.1		
			
C7—C1—C2—C3	−0.1 (5)	C2—C1—N1—O1	163.3 (3)
N1—C1—C2—C3	179.8 (3)	C7—C1—N1—O1	−16.8 (5)
C1—C2—C3—C5	−2.6 (5)	C12—C13—N2—C9	3.2 (6)
C1—C2—C3—C4	179.0 (3)	C14—C13—N2—C9	−173.9 (3)
C5—C3—C4—O4	−4.9 (5)	C12—C13—N2—Zn1	−169.7 (3)
C2—C3—C4—O4	173.6 (3)	C14—C13—N2—Zn1	13.2 (4)
C5—C3—C4—O3	175.8 (3)	C10—C9—N2—C13	−0.4 (6)
C2—C3—C4—O3	−5.7 (5)	C10—C9—N2—Zn1	171.7 (3)
C2—C3—C5—C6	2.4 (5)	O6—Zn1—N2—C13	163.0 (3)
C4—C3—C5—C6	−179.1 (3)	O7—Zn1—N2—C13	74.6 (3)
C3—C5—C6—C7	0.4 (6)	N3—Zn1—N2—C13	−14.5 (3)
C3—C5—C6—C8	−177.6 (3)	O8—Zn1—N2—C13	−107.4 (3)
C2—C1—C7—C6	2.9 (5)	O6—Zn1—N2—C9	−9.2 (3)
N1—C1—C7—C6	−177.0 (3)	O7—Zn1—N2—C9	−97.7 (3)
C5—C6—C7—C1	−3.0 (5)	N3—Zn1—N2—C9	173.2 (3)
C8—C6—C7—C1	175.0 (3)	O8—Zn1—N2—C9	80.3 (3)
C5—C6—C8—O6	−152.1 (4)	C17—C18—N3—C14	−0.1 (6)
C7—C6—C8—O6	29.9 (5)	C17—C18—N3—Zn1	−176.3 (3)
C5—C6—C8—O5	27.7 (6)	C15—C14—N3—C18	−2.6 (5)
C7—C6—C8—O5	−150.3 (4)	C13—C14—N3—C18	172.7 (3)
N2—C9—C10—C11	−2.1 (7)	C15—C14—N3—Zn1	174.0 (3)
C9—C10—C11—C12	1.6 (8)	C13—C14—N3—Zn1	−10.7 (4)
C10—C11—C12—C13	1.0 (8)	O7—Zn1—N3—C18	99.8 (3)
C11—C12—C13—N2	−3.6 (7)	O8—Zn1—N3—C18	−75.2 (3)
C11—C12—C13—C14	173.2 (4)	N2—Zn1—N3—C18	−170.4 (3)
N2—C13—C14—N3	−1.7 (5)	O9—Zn1—N3—C18	11.9 (3)
C12—C13—C14—N3	−178.7 (4)	O7—Zn1—N3—C14	−76.5 (2)
N2—C13—C14—C15	173.5 (3)	O8—Zn1—N3—C14	108.5 (2)
C12—C13—C14—C15	−3.5 (6)	N2—Zn1—N3—C14	13.3 (2)
N3—C14—C15—C16	3.0 (6)	O9—Zn1—N3—C14	−164.4 (2)
C13—C14—C15—C16	−171.9 (4)	O5—C8—O6—Zn1	−5.3 (6)
C14—C15—C16—C17	−0.7 (6)	C6—C8—O6—Zn1	174.5 (2)
C15—C16—C17—C18	−1.9 (6)	O7—Zn1—O6—C8	−135.1 (3)
C16—C17—C18—N3	2.4 (6)	O8—Zn1—O6—C8	39.5 (3)
C2—C1—N1—O2	−16.1 (5)	N2—Zn1—O6—C8	135.5 (3)
C7—C1—N1—O2	163.8 (4)	O9—Zn1—O6—C8	−47.2 (3)

### Hydrogen-bond geometry (Å, °)

**Table:**

*D*—H···*A*	*D*—H	H···*A*	*D*···*A*	*D*—H···*A*
O7—H2W···O3^i^	0.84	1.96	2.776 (4)	165
O7—H1W···O10^ii^	0.84	1.78	2.607 (4)	168
O8—H3W···O5	0.84	1.94	2.715 (4)	153
O8—H4W···O3^iii^	0.84	1.89	2.721 (4)	172
O9—H5W···O3^iv^	0.84	1.94	2.727 (4)	156
O9—H6W···O5^v^	0.84	2.57	3.414 (4)	180
O10—H8W···O4^vi^	0.84	1.79	2.631 (4)	180
O10—H7W···O5	0.84	1.87	2.713 (5)	180

Symmetry codes: (i) *x*+1, *y*−1, *z*; (ii) *x*+1, *y*, *z*; (iii) *x*, *y*−1, *z*; (iv) −*x*+1, −*y*+2, −*z*+2; (v) −*x*+1, −*y*+1, −*z*+2; (vi) −*x*, −*y*+2, −*z*+2.
